# Dermatofibrosarcoma Protuberans: A Rare Anatomical Location of Dermal Sarcoma

**DOI:** 10.7759/cureus.93809

**Published:** 2025-10-04

**Authors:** Chrysovalanti Oikonomidi, Eirini Lagogianni, Dimitrios Filippou, Dimosthenis Chrysikos

**Affiliations:** 1 Department of Dermatology, University of Athens, Athens, GRC; 2 Department of Anatomy and Surgery, School of Medicine, National and Kapodistrian University of Athens, Athens, GRC; 3 Department of Anatomy, School of Medicine, National and Kapodistrian University of Athens, Athens, GRC

**Keywords:** cd34 positive tumor, col1a1-pdgfb fusion gene, dermatofibrosarcoma protuberans, dfsp, rare skin cancer, skin sarcoma, wide local excision

## Abstract

Dermatofibrosarcoma protuberans (DFSP) is a rare, slow-growing cutaneous sarcoma that presents diagnostic and therapeutic challenges. This case report refers to a 47-year-old female patient who presented to the dermatologist with a stable, asymptomatic, firm lesion located on the proximal lower extremity. Local surgical excision was performed, and the specimen was sent for biopsy, where histopathological analysis, along with immunohistochemistry, confirmed the diagnosis of DFSP. Fluorescence in situ hybridization (FISH) analysis was also conducted, serving a supportive role in the detection of the tumor by confirming the presence of a COL1A1-PDGFB (collagen type I alpha 1 chain-platelet-derived growth factor beta) fusion gene, which is found in the majority of affected patients. As the initial excision left close margins, a second one was performed with subsequent skin grafting, and the final histopathology confirmed clear borders.

The following clinical case highlights the necessity of prompt diagnosis in skin lesions that often mimic benign conditions but are, in fact, locally aggressive. It further underscores the paramount importance of adequate surgical excision with histologically healthy margins, as recurrence rates differ significantly based on the surgical technique; more specifically, after wide local excision (WLE), the possibility of relapse falls to 4%-20%, compared to cases with narrow or positive margins, which are associated with even higher rates. This emphasizes the need to secure clear margins after the final excision, along with a structured follow-up to minimize recurrence and improve patient outcomes.

## Introduction

Dermatofibrosarcoma protuberans (DFSP) is a rare dermal sarcoma, accounting for less than 1% of all malignancies. It is slow-growing and locally aggressive in nature, and it may mimic the clinical appearance of a benign skin lesion. Most commonly, DFSP occurs in young to middle-aged adults, and although men and women are often described as being at equal risk, epidemiological data indicate slight variations across different scientific studies, with reports of approximately 53% female versus 47% male and approximately 57% male versus 43% female, respectively [1,2]. The tumor typically arises on the trunk, followed by the proximal extremities and head and neck regions, while atypical locations can introduce diagnostic and therapeutic challenges. Despite its infrequent metastatic potential, early and accurate diagnosis with histopathological and immunohistochemical evaluation is critical due to the high propensity for misdiagnosis, as DFSP often remains asymptomatic and has a high local recurrence rate if not adequately managed.

In this case report, we present a 47-year-old female patient with DFSP on the proximal right lower extremity, with no significant medical history. The aim is to emphasize the diagnostic complexity of the tumor, as well as to outline both its medical and surgical management. 

## Case presentation

**Figure 1:**
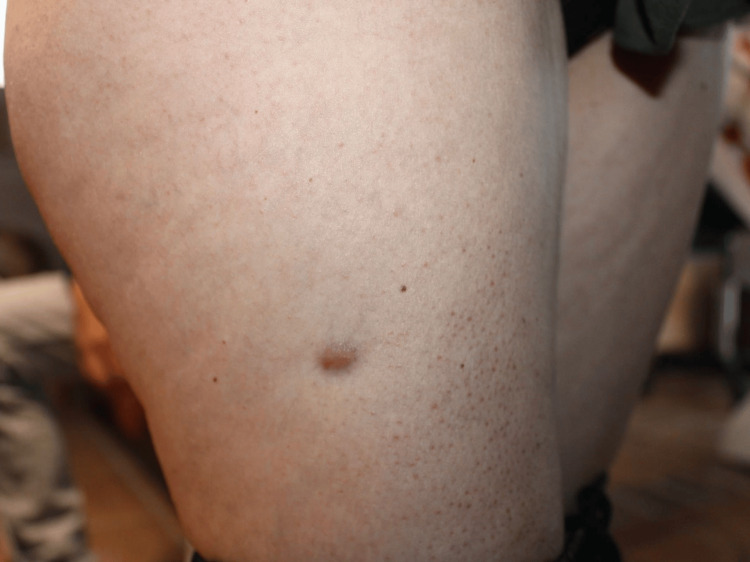
Macroscopic appearance of the lesion initially examined by the dermatologist, measuring 0.7 × 0.4 cm, with a reddish-brown coloration. A well-circumscribed, firm, protuberant lesion that was fixed and slightly elevated, with no signs of peripheral erythema, telangiectasia, ulceration, or bleeding.

A 47-year-old female patient presented to the dermatologist with an asymptomatic skin lesion located on the proximal right thigh, about 12 cm inferior to the inguinal ligament and 3 cm lateral to the femoral axis (Figure 1). It had first appeared with an unaltered morphology one year prior to the examination, remaining stable in size and color. The patient did not report any pain, pruritus, ulceration, or discharge, and she did not recall any prior trauma or insect bite in that specific anatomical location. Apart from allergic rhinitis, the patient did not have any comorbidities or a significant medical history, and no prior history of dermatofibromas or cutaneous cancerous lesions. Family and social history were negative for relevant findings. Clinical examination revealed a well-circumscribed, firm, protuberant lesion that was also fixed and slightly elevated, with no signs of peripheral erythema, telangiectasia, ulceration, or bleeding. In addition to the macroscopic description, it had a diameter of 0.7 × 0.4 cm and a reddish-brown pigmentation.

**Figure 2:**
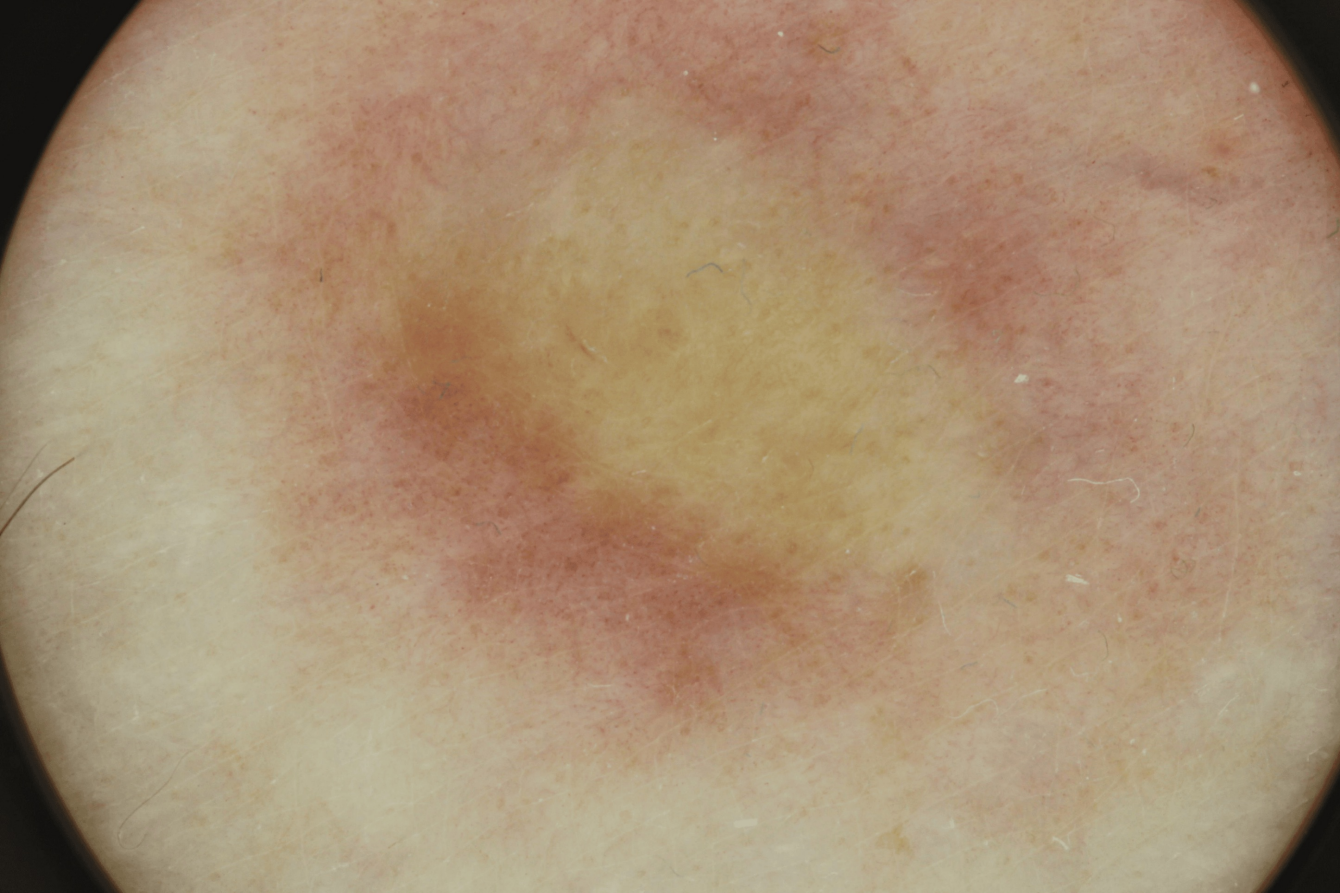
Dermoscopic examination of the lesion. A lightly pigmented network surrounded central structureless yellow-brown and hypopigmented areas, with overlying arborizing vessels - characteristics that can be found in several differential diagnoses, including DFSP, dermatofibroma, keloid, amelanotic melanoma, and non-pigmented basal cell carcinoma. DFSP, Dermatofibrosarcoma protuberans

The dermatologist proceeded to dermoscopic examination (Figure 2), which revealed a lightly pigmented network surrounding central structureless yellow-brown and hypopigmented areas, with overlying arborizing vessels. Although these findings were not pathognomonic, they raised suspicion for a range of differential diagnoses - aside from the diagnostic hypothesis of DFSP - including dermatofibroma, keloid, amelanotic melanoma, and non-pigmented basal cell carcinoma, each of which may present with overlapping vascular structures and pigment network disturbances. 

**Figure 3:**
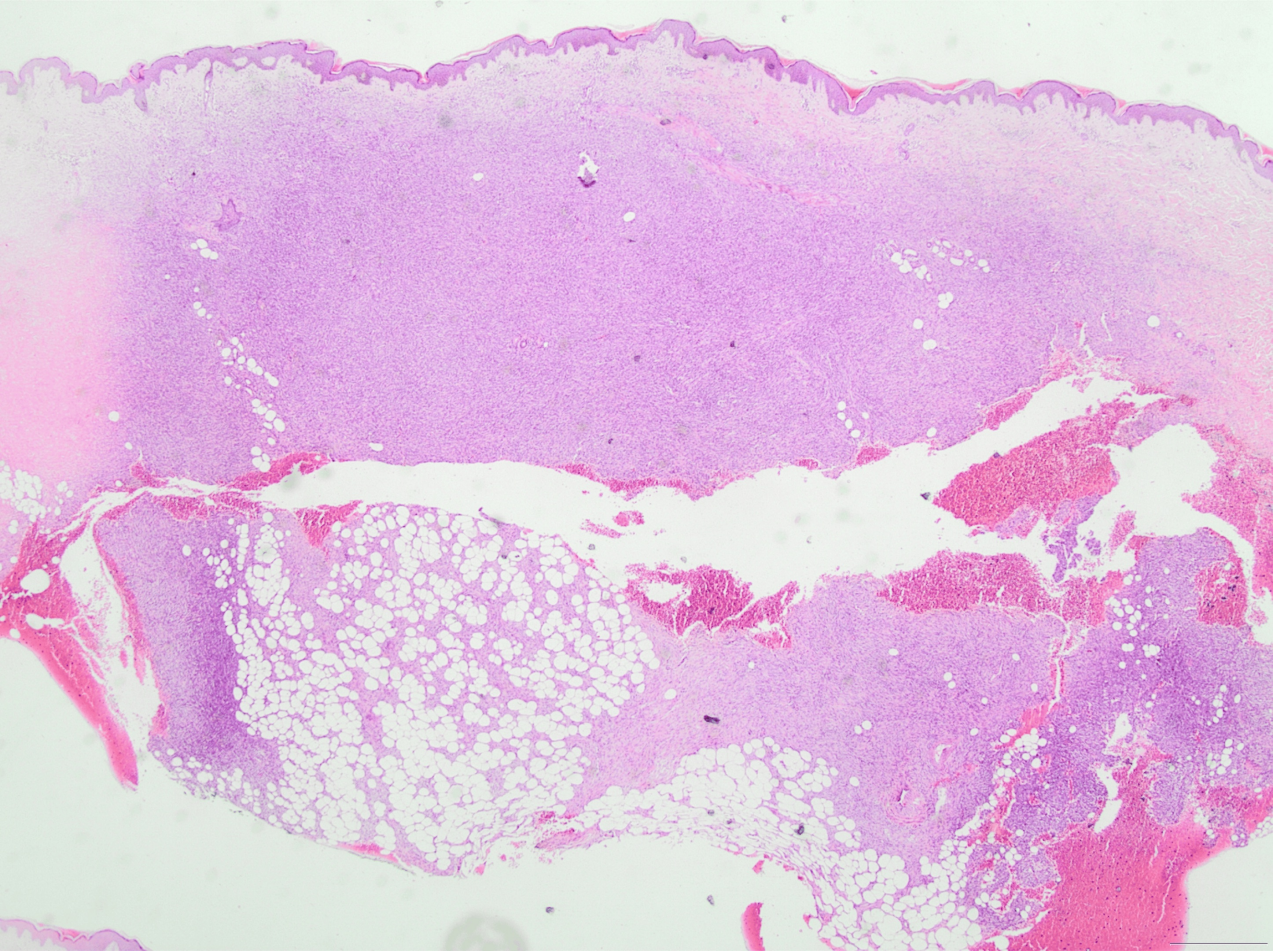
H&E stain (×20 magnification) illustrating a spindle-cell neoplasm based in the dermis and extending into the subcutaneous fat. The tumor diffuses between adipocytes, presenting the characteristic ''honeycomb'' pattern of DFSP. DFSP, Dermatofibrosarcoma protuberans

**Figure 4:**
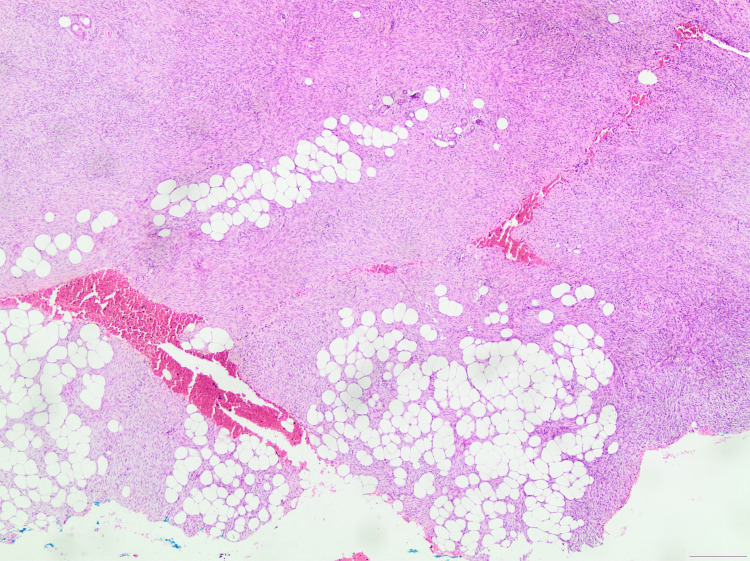
H&E stain (×40 magnification) displaying the infiltration of the neoplasm between the adipocytes, establishing the characteristic "honeycomb" pattern of DFSP. DFSP, Dermatofibrosarcoma protuberans

**Figure 5:**
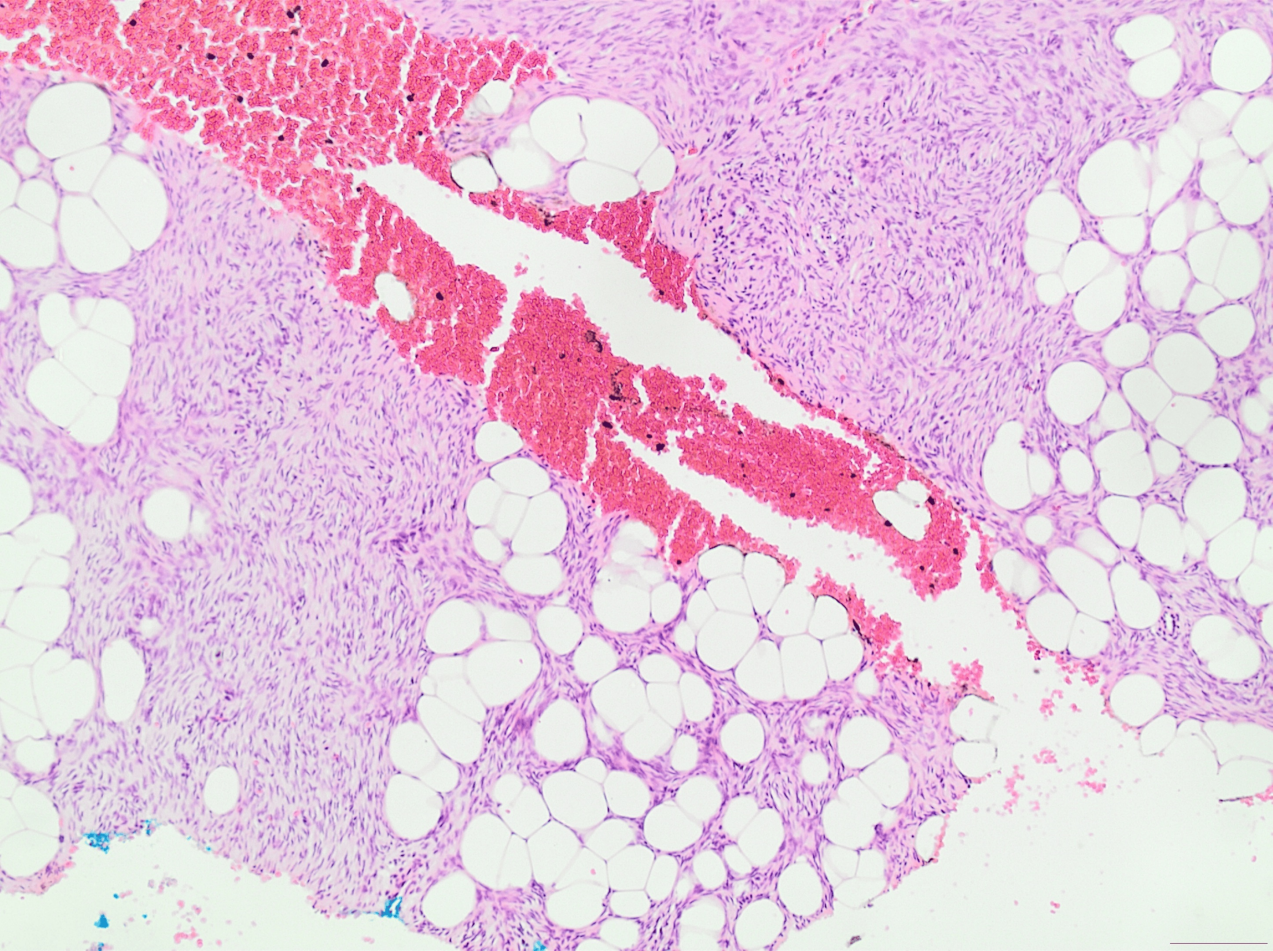
H&E stain (×100 magnification) illustrating intertwining fascicles of uniform spindle-shaped cells arranged in a storiform pattern, confirming the diagnosis of DFSP. DFSP, Dermatofibrosarcoma protuberans

**Figure 6:**
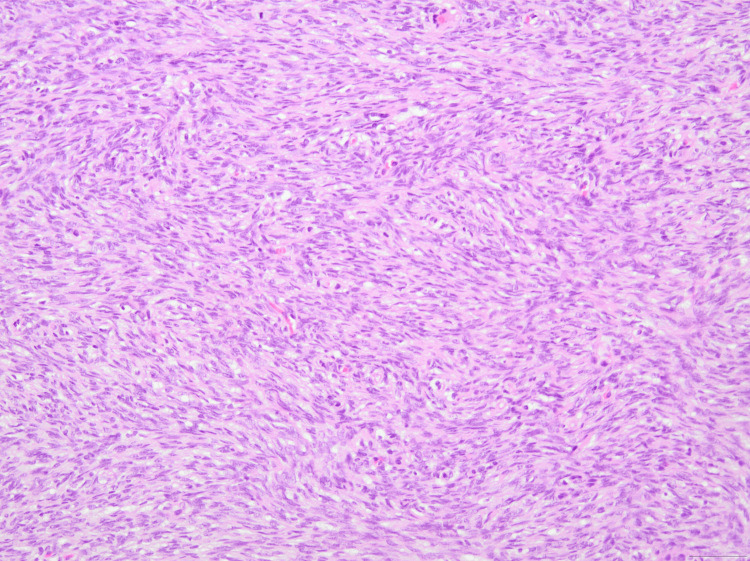
H&E stain (×200 magnification) annotating the storiform arrangement of spindle-shaped neoplastic cells, confirming the diagnosis of DFSP. DFSP, Dermatofibrosarcoma protuberans

**Figure 7:**
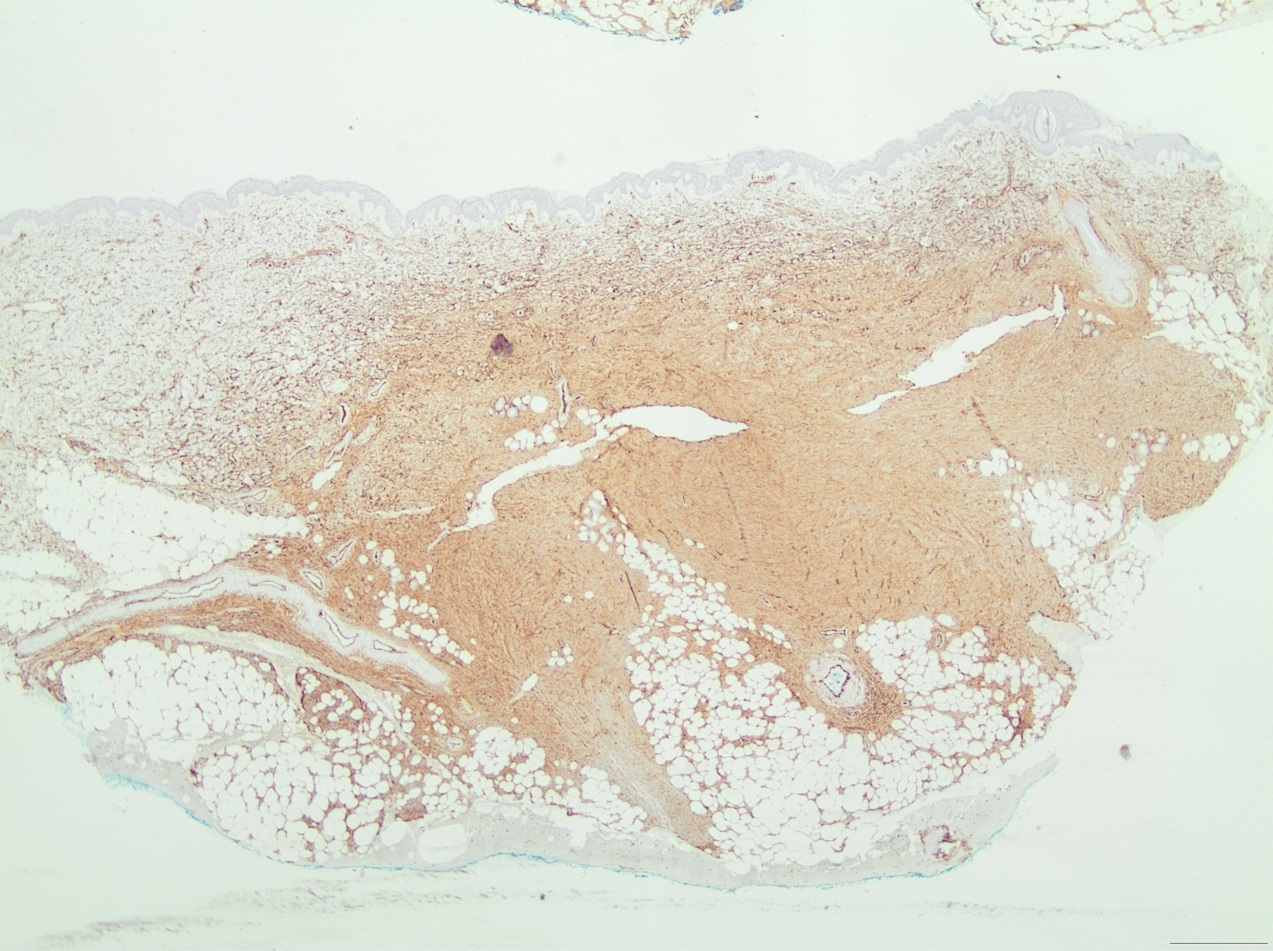
An immunohistochemical photomicrograph showing CD34 positivity at ×20 magnification.

**Figure 8:**
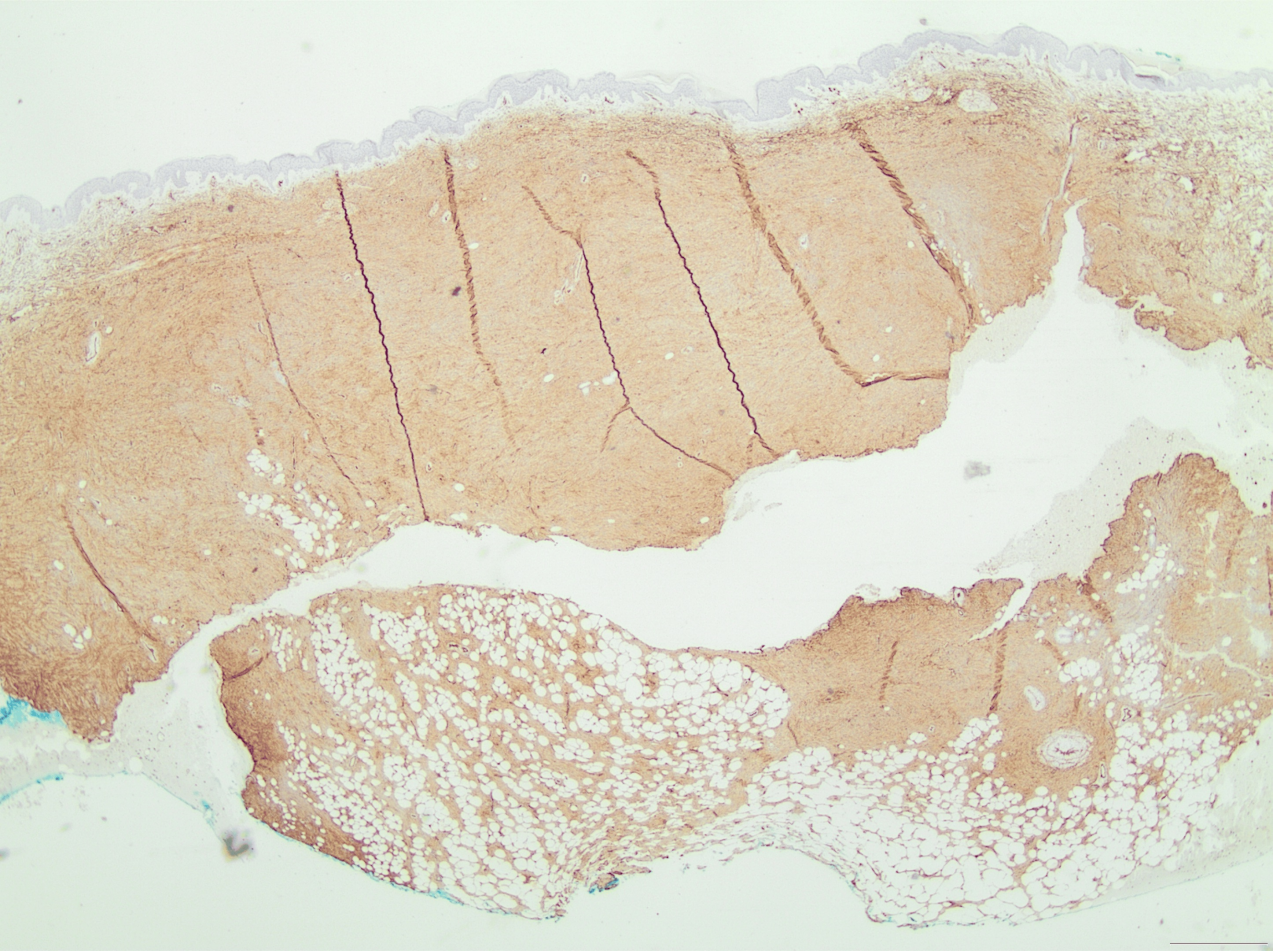
An immunohistochemical photomicrograph showing strong CD34 positivity at ×40 magnification.

**Figure 9:**
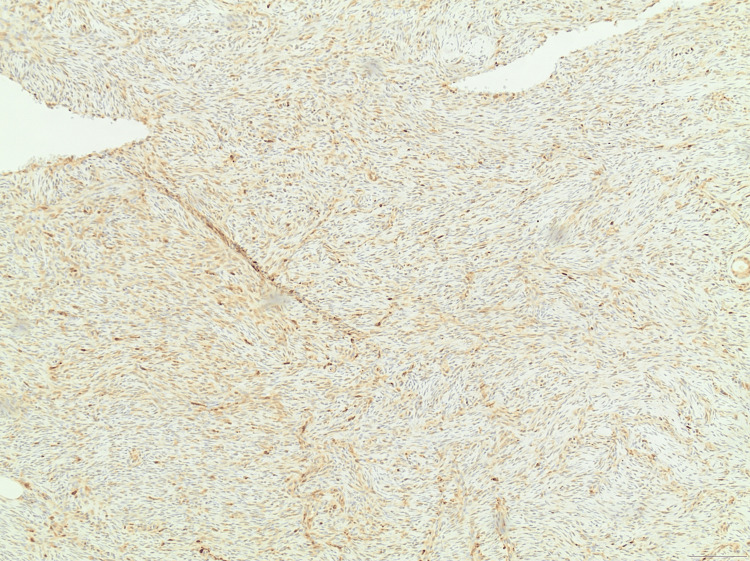
An immunohistochemical photomicrograph showing the absence of staining for Factor XIIIa at ×100 magnification, a common finding in DFSP lesions. DFSP, Dermatofibrosarcoma protuberans

Local surgical excision was performed, and the specimen was sent for biopsy to establish a definitive diagnosis. Histopathological examination confirmed the diagnosis of DFSP, with the microscopic description reporting a neoplastic lesion of the dermis, characterized by densely packed, spindle-shaped neoplastic cells arranged in a storiform (cartwheel) pattern, extending deeply into the subcutaneous fat in a "honeycomb" pattern (Figures 3-6). Mitotic activity of the lesion was low (about 3-4 mitoses per 10 high-power fields), a finding compatible with the typically slow-growing but locally aggressive biological behavior of DFSP [2]. Immunohistochemically, the neoplastic cells were positive for CD34 and negative for FXIIIa markers (Figures 7-9). As both the peripheral and deep surgical margins were close to the tumor, wider excision was recommended by the histopathologist. Furthermore, a fluorescence in situ hybridization (FISH) analysis was performed on formalin-fixed, paraffin-embedded biopsy tissue using the SPEC COL1A1/PDGFB Dual Color Dual Fusion Probe (ZytoVision, Bremerhaven, Germany), as the COL1A1-PDGFB (collagen type I alpha 1 chain-platelet-derived growth factor beta) gene fusion is observed in the vast majority of DFSP cases. Consequently, out of a total of 100 interphase nuclei analyzed with the probe, 55 showed abnormal hybridization patterns (55%) - a finding that provides strong molecular confirmation of the diagnosis, as most cytogenetics laboratories consider a threshold of >10% abnormal nuclei sufficient for a positive result. 

**Figure 10:**
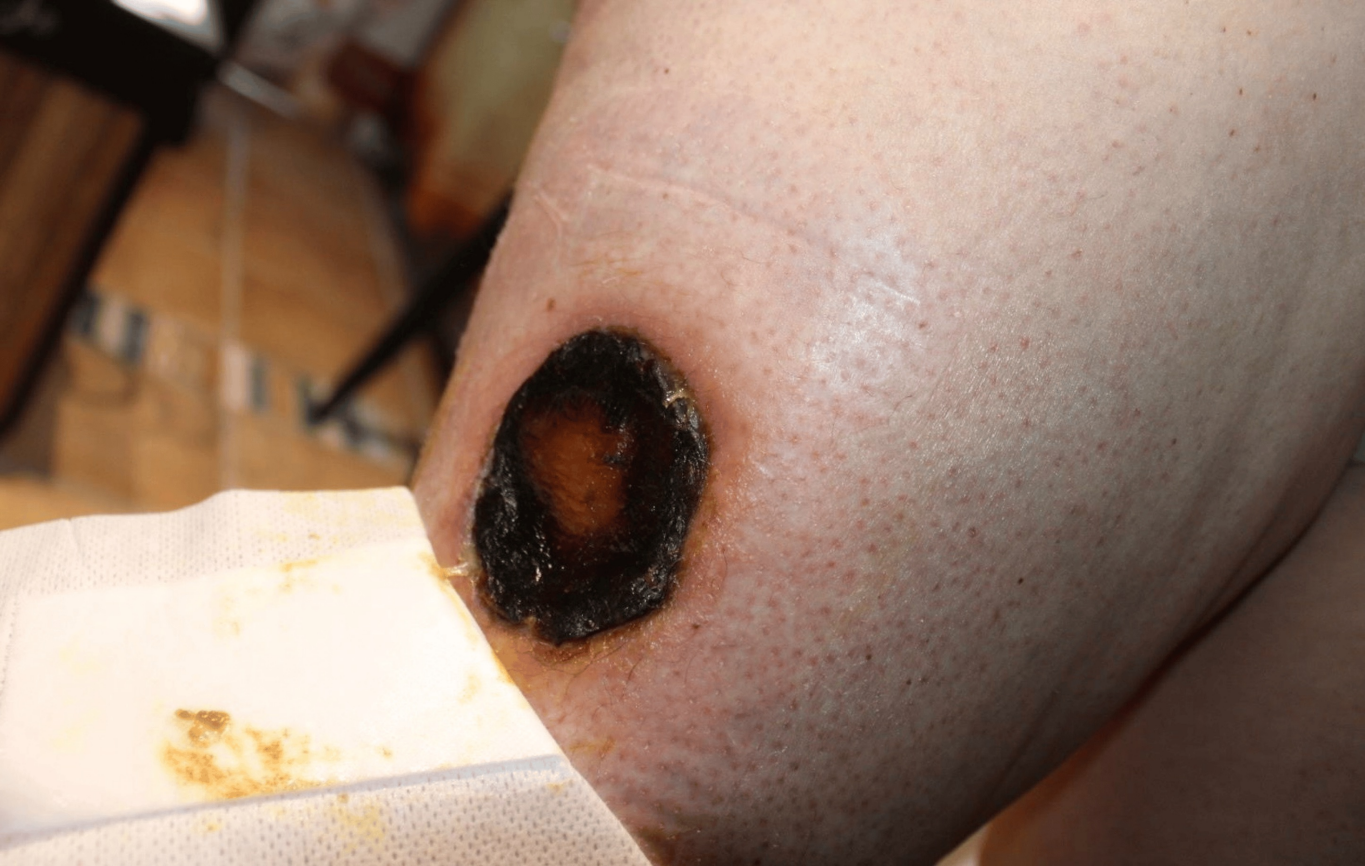
Gross view of the surgical site following wide local excision and coverage with a skin graft from the femoral region.

**Figure 11:**
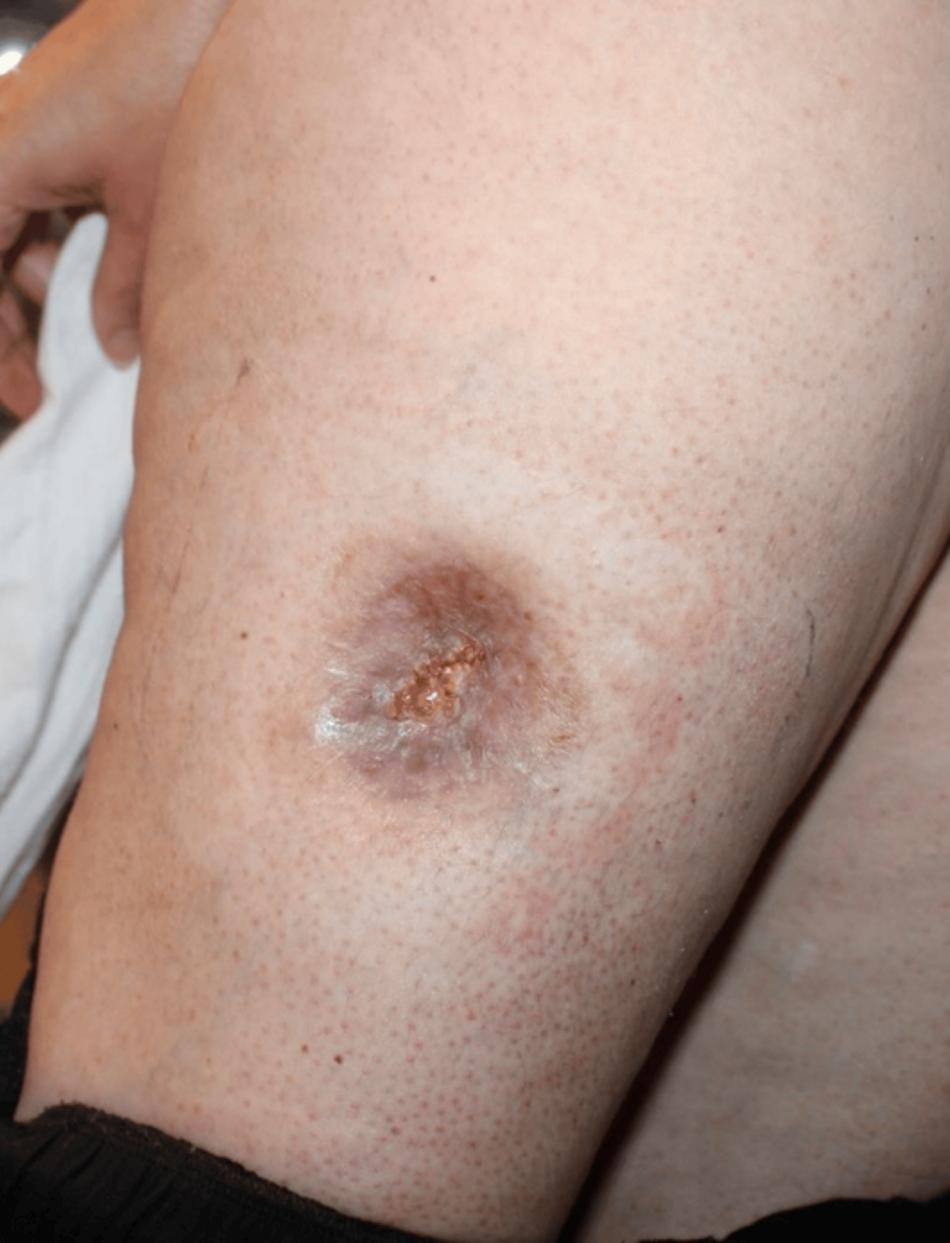
Follow-up appearance of the reconstructed site.

The patient was subsequently referred to a general surgeon for wide resection with appropriate margin control. The tissue sample measurements were 4 × 3.4 cm, with a thickness of 2 cm, exhibiting a longitudinal scar of 1.5 cm, along with a fibro-adipose tissue section measuring 2.5 × 1 × 0.5 cm. Due to the size of the defect, a full-thickness skin graft was needed from the femoral region of the patient for reconstructing the surgical wound, ensuring adequate coverage of the underlying structures (Figures 10-11). The specimen from the excised material was submitted for histopathological evaluation, revealing that the lesion had been completely resected with clear surgical margins on permanent sections. More specifically, microscopically, the scarred lesion exhibited focal disruption of the epidermis and microabscess formation. In the underlying dermis near the subcutaneous adipose tissue, minimal residual elements of the previously diagnosed DFSP were established, with no evidence of neoplastic infiltration in the muscle tissue sample.

Due to the possible risk of local recurrence, the patient remains under close follow-up supervision by the dermatologist, undergoing clinical examination every six months for the first five years after diagnosis, and annually for the following 5 to 10 years.

## Discussion

DFSP is considered one of the rarest slow-evolving sarcomas of the skin, representing less than 2% of all soft tissue sarcomas and less than 1% of all cancers. It expands locally, sometimes with an aggressive behavior and an increased propensity for local recurrence after excision [3]. It usually affects individuals from the second to the fifth decade of life and does not show any gender preference. Although it most commonly arises on the trunk (50%-60%), in this case, it is noteworthy that DFSP developed on the proximal right lower extremity, which accounts for only one to four or five cases [4].

Early clinical misdiagnosis of DFSP is not uncommon, as in its early stages it may clinically mimic benign lesions such as dermatofibroma, epidermal inclusion cysts, or keloids [5]. Therefore, early referral to a dermatologist is crucial to ensure appropriate assessment, since histopathological evaluation of biopsied tissue remains the gold standard for diagnosis.

During recent decades, dermoscopy has emerged as a helpful adjunctive tool in the evaluation of cutaneous sarcomas. Although there is no particular pathognomonic dermoscopic pattern for DFSP, certain features have been recurrently observed in published cases. These encompass a pink or hypopigmented background with structureless yellowish to brown areas, a peripheral pigmented network, and prominent arborizing or linear-irregular vessels [2]. Such features were also detected in our patient and, while not diagnostic on their own, in the appropriate clinical context they raise suspicion for DFSP and prompt further histological investigation.

In this case report, the histopathology of the specimen was characteristic for the diagnosis of DFSP. A uniformity of spindle-shaped cells organized in a storiform or cartwheel pattern, penetrating the dermis and subcutaneous tissue, along with an entanglement of fat in a “honeycomb” arrangement, as well as the immunohistochemistry of the tumor - where CD34 is strongly positive and Factor XIIIa is negative - provided diagnostic confirmation [6]. These features also aid in distinguishing DFSP from dermatofibroma (which is CD34-negative and Factor XIIIa-positive), as well as from other benign or malignant spindle cell tumors.

The pathogenesis of this specific neoplasia relies on the activation of the PDGFB pathway, stimulating tumor proliferation. Therefore, DNA analysis should be strongly considered, as in this case, to search for the chromosomal translocation t(17;22)(q22;q13), since it results in the formation of a COL1A1-PDGFB fusion gene, which leads to constitutive activation of the PDGFB pathway [7]. The existence of this chromosomal translocation can also aid in treatment prognosis, as mentioned below.

Regarding treatment, the primary therapeutic approach is surgical excision, with wide local excision (WLE) with margins of 2-3 cm traditionally employed. However, Mohs micrographic surgery (MMS) has emerged as the preferred technique in many centers due to its tissue-sparing approach and superior margin control, resulting in lower recurrence rates [8]. This technique allows the surgeon to first remove the visible tumor and then excise thin surrounding layers, which are immediately examined under the microscope. If cancer cells are still present in the tissue, additional tissue is removed only from the specific area where they remain. Thus, the surgeon achieves minimization of healthy tissue loss.

Regardless of the surgical approach, there are cases of recurrent, metastatic, or even unresectable DFSP due to anatomical locations with restricted surgical margins. In these patients, targeted therapy with a tyrosine kinase inhibitor, imatinib mesylate, which blocks the PDGFB receptors, can be considered, especially in tumors refuging the COL1A1-PDGFB fusion gene [9]. Furthermore, in such cases, postoperative radiotherapy (PORT) can play a crucial role in suppression of local disease, as it has been demonstrated that it could serve as adjuvant therapy in the presence of clinicopathologic risk factors and as secondary treatment after incomplete resection due to the aforementioned reasons [10]. Notably, these interventions provide adequate long-term control when incorporated within a multidisciplinary treatment approach.

In the present case, following the initial excision revealing close surgical margins, a wider excision was performed in order to attain complete tumor elimination and minimize the possibility of relapse, with sequential histopathological evaluation confirming clear margins. Given that the risk of local tumor recurrence is found to be the lowest in cases where the tumor has been successfully excised with adequate borders [5], the patient was placed on a structured protocol of follow-up visits with dermatological examinations every six months for the first five years, followed by annual visits thereafter [11]. In addition, local recurrence rates after MMS fall to approximately 1%-2%, compared to those after WLE, which fall to 4%-20%, with even higher rates when associated with narrow or positive margins [5]. 

As a final point, this case report underscores the necessity of prompt diagnosis, thorough dermatological and histopathological assessment, adequate surgical excision, and long-term follow-up in order to achieve optimal outcomes for candidates with clinical suspicion of DFSP.

## Conclusions

In conclusion, this case report emphasizes the importance of recognition and thorough diagnostic evaluation for DFSP, particularly when presenting in less common anatomical areas and in patients without any substantial present, past, or family medical history. Furthermore, the necessity of a multidisciplinary approach is highlighted in order to diagnose and manage effectively this type of cutaneous sarcoma. Although not diagnostic in isolation, dermatological examination with dermoscopy is of paramount importance as it raises initial suspicion of DFSP, allowing the physician to perform an early biopsy, which will be complemented by histopathology, ensuring the definitive diagnosis. In addition, immunohistochemistry and molecular analysis (COL1A1-PDGFB fusion analysis) not only support the diagnosis but also contribute to guiding possible targeted therapies, especially in advanced or recurrent cases.

The patient described in this report underwent two WLEs in order to achieve histologically clear margins, aiming to minimize the risk of tumor relapse. Despite that, MMS may be optimal in some selected cases; it is not universally employed. Last but not least, given the tumor’s potential for local recurrence even after adequate excision, long-term follow-up is crucial to achieving the best possible outcome for patients. All things considered, this case provides clinical awareness by presenting a less common anatomical appearance of DFSP and reinforcing the necessity of systematic, evidence-based management. 
